# S2. CHILDHOOD TRAUMA IS ASSOCIATED WITH SEVERITY OF AT-RISK MENTAL STATE AND PSYCHOSIS IN UHR INDIVIDUALS AND PATIENTS WITH SCHIZOPHRENIA

**DOI:** 10.1093/schbul/sby018.789

**Published:** 2018-04-01

**Authors:** Attilio Rapisarda, Oon Him Peh, Kang Sim, Jimmy Lee

**Affiliations:** Institute of Mental Health

## Abstract

**Background:**

Experiences of abuse during childhood are highly prevalent in psychiatric populations and are associated with a higher risk of developing psychosis during adolescence and young adulthood. This study explored the association between childhood trauma, psychotic symptoms and comorbid symptomatology in Ultra-High Risk individuals and schizophrenia patients.

**Methods:**

59 patients diagnosed with schizophrenia (mean age= 33), 164 individuals at Ultra-High Risk for psychosis (UHR) (mean age= 22) and 60 healthy individuals (mean age= 28) were recruited from the clinic and the community and assessed using the Structured Clinical Interview for DSM-IV (SCID), Comprehensive Assessment of At-Risk Mental State (CAARMS), Global Assessment of Functioning (GAF) and Childhood Trauma Questionnaire. Patients and UHR were also assessed on the Positive and Negative Syndrome Scale (PANSS) and Beck Anxiety Inventory (BAI). A Kruskal-Wallis one-way analysis was performed to detect significant differences in the severity of reported trauma across groups. Multiple regression analyses were computed on Blom-ranked, normalized scores to measure the association between reported trauma and symptomatology in the UHR participants and patients.

**Results:**

Significant differences in severity of reported childhood traumatic experiences were found across the three groups for emotional abuse (p= .000), emotional neglect (p= .000), physical neglect (p= .000), physical abuse (p= .001) and sexual abuse (p= .007). The severity of traumatic experiences reported by patients and UHR individuals was consistently higher compared with controls, with effect sizes ranging from a minimum of Cohen’s d= 0.54 (sexual abuse) to a maximum of Cohen’s d= 0.97 (emotional abuse).

In UHR, higher CTQ total scores, reflecting more severe overall abuse, were associated with more severe prodromal psychotic symptoms (β= .259, p= .001). Also, more severe anxiety symptoms were associated with more severe emotional (β= .365, p= .000), physical (β= .283, p= .000), and sexual abuse (β= .214, p= .006).

In UHR and patients, higher PANSS negative sub-scale scores were associated with higher levels of emotional (β= .196, p= .012) and physical neglect (β= .175, p= .026).

**Discussion:**

Our findings support previous evidence on the higher prevalence of different types of abuse experienced during childhood by schizophrenia patients and individuals at risk for psychosis. Among the different types of abuse investigated, severe emotional abuse is most strongly associated with severe prodromal symptomatology and anxiety experienced during young adulthood by UHR individuals.

